# Recent Versus Old Previous Injury and Its Association with Running-Related Injuries During Competition by SeRUN® Running Profiles: a Cross-sectional Study

**DOI:** 10.1186/s40798-018-0164-x

**Published:** 2018-11-12

**Authors:** Jaime Leppe, Manuela Besomi

**Affiliations:** School of Physical Therapy, Universidad del Desarrollo, Avenida La Plaza 680, Las Condes, Santiago Chile

**Keywords:** Running, Marathon, Aetiology, Injury

## Abstract

**Background:**

Previous injury in the last 12 months is the main risk factor for future running-related injuries (RRI) during training and competition environments. However, the relationship between a recent versus old previous injury and a new RRI has not been established yet, nor a separate analysis by different types of runners.

**Methods:**

An online questionnaire was sent to 6000 participants of a running event (10 km, 21 km and 42 km), 10 days following the event. The questionnaire included the following information: the presence and topography of new RRIs during the race, old previous injury (from 12–4 months before the race), recent previous injury (from 3–0 months before the race), running experience, training factors and socio-demographic characteristics. Univariate binomial regression analysis was applied to assess different associated factors, and multivariable binomial backward regression (*p* < 0.05) was used to analyse the relationship between the new and previous injury.

**Results:**

A total of 868 surveys were analysed (10 km, 32.6%; 21 km, 52%; 42 km, 15.4%). The median age was 38 years (IQR 31–46), and 63.5% were males. Previous injury was reported by 30.3% and 27.6% for old and recent, respectively. The majority of runners were categorised into the advanced group (42.9%), having more than 5 years of running experience. During the race, 7.0% reported a RRI, with 36.1% located at the knee. The multivariable analysis showed an association only between new injury and recent injury.

**Conclusion:**

The delineation of recent and old previous injuries should be considered in running epidemiological research.

## Key Points

- Previous injury in the last 12 months still remains the main associated factor with running-related injuries in epidemiological studies of running.
- Epidemiological studies in running research should incorporate the delineation of recent and old injuries.
- Clinicians should consider different management strategies of race expectations and training planning when an injured runner is close to a competition, especially when it is a recent injury.

## Background

Previous injury in the last 12 months is the main risk factor for future running-related injuries (RRIs) during training and competition situations [1]. A subsequent injury is defined as any injury following an initial (index) injury [2]. The relationship between previous injury and subsequent lower limb injury is poorly understood in runners; however, it has been studied in sports populations, such as rugby, soccer and football [3]. The updated subsequent injury categorisation (SIC-2.0) model [4] provides a valid framework for accurate categorisation of subsequent injuries with eight mutually exclusive subsequent injury categories. In a systematic review, Toohey et al. [3] found that a history of lower limb muscular or joint injuries was associated with a variety of lower limb subsequent injuries that are of a different type. These findings suggest that the relationship between previous injury and subsequent injuries is complex because multiple determinants and factors are involved. This relationship needs to be considered when planning prevention strategies, as the ability to adapt the body to a particular load (running participation) might be influenced by the capacity of the tissues to tolerate that load [5]. To the extent of the authors’ knowledge, only one prospective study has considered the classification of previous injury into less than 3 months, between 3 and 12 months, and more than 12 months, for studying its association with a new injury [6]. Even though the study prospectively followed a cohort of female runners training for a 5 km or 10 km race, participants were retrospectively asked about the presence of a previous injury. The relationship between a recent versus old previous injury and the occurrence of a new RRI during competition has not been established yet, nor a separate analysis by different types of runners. The aim of this study was to determine the relationship between a recent and old previous injury and the presence of RRIs during competition among different types of runners.

## Methods

An online questionnaire was sent to 6000 participants of a running event (Maratón de Viña del Mar) held in Viña del Mar, Chile, in October 2, 2017. An email invitation was distributed by the race organisers 10 days following the event to all registered runners who participated in one of three different distances (10 km, 21 km and 42 km). Individuals were eligible to participate if they were 18 years or older and had competed in one of the three running distances. Informed consent was confirmed when individuals clicked on the survey link, thus authorising investigators to use their de-identified data for further analysis. This study was performed in accordance with the standards of ethics outlined in the Declaration of Helsinki and approved by the Research Ethic Committee at Universidad del Desarrollo, no. 2017-105. The questionnaire included the following information: presence and topography of RRIs during competition (new), old previous injury (from month 12th–4th before the race), recent previous injury (from month 3rd–0 before the race), SeRUN® running profiles (years of running experience), training factors (weekly mileage for weeks 1, 2, 3 and 4 prior to the competition; hours and frequency of running training), race distance (10 km, 21 km, or 42 km) and socio-demographic characteristics. According to a previous study [7], SeRUN® running profiles were classified as follows: Beginner, Basic, Middle and Advanced. According to the weekly mileage variable, acute volume (week prior to competition), chronic volume and acute chronic workload ratio (coupled ACWR) [8] were calculated. For the ACWR calculation, only external load (weekly volume) was used; acute volume included mileage of the last week plus race distance; chronic volume was calculated as the average of weeks 1, 2, 3 and acute week (coupled). ACWR cut-off values of < 0.85, between 0.85–1.35 and > 1.35 were used to investigate the injury risk differences between them [5, 9]. A running-related injury was defined as “running-related (training or competition) musculoskeletal pain in the lower limbs that causes a restriction on or stoppage of running (distance, speed, duration, or training) for at least 7 days or 3 consecutive scheduled training sessions, or that requires the runner to consult a physician or other health professional.” [10], which was used for all types of injuries studied (old and recent previous injury and injury during competition). The report of previous and new injuries was collected as a binary (injured/not injured) variable, and participants could report the topography for each injury.

A Shapiro-Wilk test was used to evaluate whether all quantitative variables were normally distributed. Comparisons of training factors, as weekly mileage (chronic, acute and ACWR), frequency and hours of training between injured and non-injured participants were performed for each distance and running profile using the Mann-Whitney *U* test for non-parametric data and the chi-square test for ordinal data. Univariate binomial regression analysis was applied to assess different control variables (i.e. sex, age, BMI, weekly running volume and ACWR), and multivariable binomial backward regression (*p* < 0.05) was used to analyse the relationship between the new and previous injury, adjusted for potential control variables by SeRUN® running profiles. All statistical analyses were performed using 13.0 STATA software.

## Results

A total of 868 surveys were analysed (10 km, 32.6%; 21 km, 52%; 42 km, 15.4%), with a response rate of 14.5%. The median age of participants was 38 years (IQR 31–46), and 63.5% were males. The distribution of running profiles was Beginner (3.6%), Basic (29.6%), Middle (24%) and Advanced (42.9%). Previous injury was reported by 263 (30.3%) and 240 (27.6%) participants for old and recent, respectively, where the knee and foot/ankle segments were the most common sites injured. The characteristics of participants by race distance can be found in Table 1.

**Table 1:** Description of socio-demographic, health characteristics and running practice based on the registered distance

	Total		10 km		21 km		42 km	
	*n* = 868		*n* = 283		*n* = 451		*n* = 134	
Age (years)^a^	38	(31–46)	37	(29–47)	38	(31–45)	40	(35–47)
Gender (male)^b^	551	(63.5)	136	(48.1)	300	(66.5)	115	(85.8)
Educational level^b^								
Under 8 years	31	(3.7)	8	(2.9)	20	(4.6)	3	(2.2)
Between 8 and 12 years	62	(7.3)	21	(7.7)	31	(7.1)	10	(7.5)
Between 12 and 18 years	299	(35.4)	96	(35.2)	155	(35.4)	48	(35.8)
Over 18 years	453	(53.6)	148	(54.2)	232	(53.0)	73	(54.5)
BMI (kg/m^2^)^b^	24.4	(22.8–26.2)	24.7	(22.9–26.6)	24.4	(22.7–26.3)	24.2	(22.8–25.6)
Recent previous injury (3rd–0 month)	240	(27.6)	89	(31.4)	125	(27.7)	26	(19.4)
Old previous injury (12th–4th month)	263	(30.3)	67	(23.7)	162	(35.9)	34	(25.4)
Sleeping hours^b^								
Under 5 h	9	(1.1)	4	(1.5)	4	(0.9)	1	(0.7)
Between 5 and 6 h	174	(20.6)	55	(20.1)	90	(20.5)	29	(21.6)
Between 6 and 7 h	380	(45)	121	(44.3)	199	(45.4)	60	(44.8)
Between 7 and 8 h	257	(30.4)	88	(32.2)	133	(30.4)	36	(26.9)
Over 8 h	25	(3)	5	(1.8)	12	(2.7)	8	(6)
N° of previous participation in competition^a^	5	(3–8)	4	(2–7)	5	(3–8)	6	(4–10)
Training plan^b^								
Coach	293	(35.5)	68	(25.6)	163	(38.2)	62	(46.6)
Mobile application	232	(28.1)	98	(36.8)	114	(26.7)	20	(15)
Self-administered	301	(36.4)	100	(37.6)	150	(35.1)	51	(38.3)
SeRUN® running profile^b^								
Beginner (< 1 year)	31	(3.6)	23	(8.1)	7	(1.6)	1	(0.7)
Basic (1–2 years)	257	(29.6)	124	(43.8)	118	(26.2)	15	(11.2)
Middle (3–4 years)	208	(24)	61	(21.6)	115	(25.5)	32	(23.9)
Advanced (≥ 5 years)	372	(42.9)	75	(26.5)	211	(46.8)	86	(64.2)

^a^Median (interquartile range); ^b^absolute frequency (%); *BMI* body mass index, *km* kilometres, *m* metres, *kg* kilogrammes

During the race, 61 participants (7.0%) reported a RRI, with 36.1% located at the knee. By running profile new injury was 12.9%, 5.8%, 7.2% and 7.3% for Beginner, Basic, Middle and Advanced, respectively. From those participants who reported an injury during the race, 19 runners (31.2%) had also reported an old and recent previous injury. Among running profiles, a higher proportion of old previous injury was found in Advanced runners (34.1%); recent previous injury in Basic (32.3%); and new injury in Beginner (12.9%). Weekly mileage (acute, chronic, ACWR) was not significant different (*p* > 0.05) between injured and non-injured runners, analysing the data as overall, by race distance or running profiles. Table 2 shows the comparison of training characteristics and acute chronic workload ratio (ACWR) by registered distance.

**Table 2:** Comparison of training characteristics and acute chronic workload ratio (ACWR) by registered distance

	Total		10 km		21 km		42 km	
	*n* = 868		*n* = 283		*n* = 451		*n* = 134	
Hours of running training (h/week)^a^	4	(3–6)	3	(2–4)	4	(3–6)	5.5	(4–7)
Frequency of running training	3	(3, 4)	3	(2, 3)	3	(3, 4)	4	(3–5)
Chronic volume (km/week)^c^	24	(15.3–38.5)	14.5	(9.8–21.3)	27.3	(19–37.3)	50.5	(37.5–63.8)
Week 1	20	(12–37.5)	13	(8–20)	22	(15–37)	47	(34–65)
Week 2	20	(10–36.5)	12	(6–20)	22	(12–36)	48.5	(30–63)
Week 3	20	(10–35)	12	(7–20)	22	(15–35)	44	(21–60)
Week 4	15	(9–25)	10	(6–17)	15	(10–25)	20	(10–30)
Acute volume (km/week)^c^	35	(25–51)	20	(16–27)	36	(31–46)	62	(52–72)
ACWR	1.4	(1.2–1.7)	1.5	(1.2–1.8)	1.4	(1.2–1.7)	1.3	(1.1–1.5)
ACWR category								
< 0.85	40	(4.6)	22	(7.8)	15	(3.3)	3	(2.2)
0.85–1.35	344	(39.6)	91	(32.2)	178	(39.5)	75	(56)
> 1.35	484	(55.8)	170	(60.1)	258	(57.2)	56	(41.8)

^a^Median (interquartile range); ^b^absolute frequency (%). *km* kilometres, *m* metres

^c^Acute volume is the sum of week 4 + race distance; chronic volume is the average of week 1, 2, 3 and acute volume

The univariate analysis revealed a significant association between new injury and old (OR = 1.92 [CI 95% 1.13–3.26], *p* = 0.015) and recent previous injuries (OR = 3.95 [CI 95% 2.32–6.72], *p* < 0.001), but no association was found with any control variables (i.e. age, sex, BMI, weekly running volume and ACWR). By profile, the univariate analysis was significant (*p* < 0.05) for recent injury in Basic, Middle and Advanced runners (Table 3). The odds ratio of presenting double previous injury (old and recent) was OR = 4.8 (CI 95% 2.42–9.40, *p* < 0.01) compared with OR = 2.7 (CI 95% 1.43–5.1] *p* = 0.02) of those reporting at least one (old or recent), taking as reference no previous injury. However, the multivariable analysis showed an association only between new injury and recent injury (OR = 3.95 [CI 95% 2.32–6.72], *p* < 0.01), being removed from the model any sociodemographic, training, old and double previous injury variables.

**Table 3:** Univariate binomial regression analysis between new injury and recent previous injury, by SeRUN® profile

Recent previous injury	OR	95% CI	*p* value
Beginner	4.40	0.49–39.2	0.184
Basic	4.63	1.53–14.02	0.007
Middle	3.36	1.16–9.74	0.026
Advanced	4.28	1.92–9.52	< 0.01

*OR* odds ratio, *95% CI* 95% of confidence interval

## Discussion

The results of the current study revealed greater percentages of previous injury in the last 12 months (including both recent [27.6%] and old [30.3%]) compared with other studies [1, 11]. Previous injury in the last 12 months still remains the main associated factor for developing a new injury, as it is reported in other studies [1, 3]. Additionally, our findings showed a greater association for recent previous injury (the last 3 months), compared with an old previous injury when studying the presence of injury during competition. Despite the limitations of the study design, there appears to be a dose-response degree when analysing one versus two injuries. The presence of two previous injuries has higher risk for a new injury, but in a multivariable analysis, the recent injury is more powerful than having two injuries. This finding could be partially explained because of an incomplete healing process from the recent injury, exposing the tissue to a lower capacity of adaptation to training loads and finally, the race itself. Only one study [6] has analysed previous injury into different categories (e.g. less than 3 months, between 3 and 12 months, and more than 12 months) in reference to a running event (5 km and 10 km). Authors reported an association with injury only for a previous injury greater than 12 months, and weekly running distance (greater than 30 km/week). The differences in our findings might be due to the study population, study design and definition of running-related injury used. The current study included runners with a high level of running experience (mainly Advanced profile) [7], and the included race distances were higher (10 km, 21 km and 42 km). Likewise, the frequency of running-related injuries found in this study (7%) was lower than those reported in other studies [12, 13]. A relationship between a new injury and training load was not possible to establish. However, given the high experience level of the population, it is highly likely that runners with greater knowledge about how to manage their training plan can self-manage their running-related symptoms [7].

### Limitations

Although recall bias is more likely in the old question [14], asking for recent previous injury seems to have more impact than old injury in the association with a RRI during competition. Future prospective studies should investigate the interaction between old, recent and new injuries in runners, incorporating current categorisation models for subsequent and recurrent injuries [4]. Additionally, better methods of quantifying training loads should be considered to enhance data accuracy and the understanding of training load from current theoretical models, as the acute chronic workload ratio [5, 8]. A recent prospective study protocol has been published with high potential to answer these questions [15].

Additionally, we cannot ensure that runners were (or not) injury free at the time of the race or that the new injury is a non-recovered or an aggravation of the recent previous injury. However, our primary aim was to identify the relationship between a previous injury with the occurrence of a new injury during the race, which is possible when using the RRI consensus definition in retrospective studies [4]. Care should be taken when interpreting these results as a causality relationship cannot be made because of the cross-sectional nature of the study.

## Conclusion

The delineation of recent and old previous injuries should be considered in running epidemiological research. A recent previous injury has a higher association with a new injury during competition compared with an old previous injury when studying its relationship retrospectively. Clinicians could incorporate these findings into the clinical practice when evaluating runners who are injured and are training for a race. Training variables, such as mileage, frequency and intensity, must be planned and monitored between the clinician, coach and athlete due to the high likelihood of a new injury. Care should be taken with language and educational management of these potential injuries [16], and use a consensual coaching protocol that provides safety to the patient through the manipulation of training loads, and incorporating functional assessment tools reported by the patient.

## Abbreviations

ACWR: Acute chronic workload ratio
BMI: Body mass index
RRI: Running-related injuries
